# *EPAS1* targeting by miR-152-3p in Paclitaxel-resistant Breast Cancer

**DOI:** 10.7150/jca.46898

**Published:** 2020-09-02

**Authors:** Ying Song, Mo Zhang, Man Man Lu, Li Yuan Qu, Si Guang Xu, Yong Zhen Li, Ming Yong Wang, Hui Fang Zhu, Zhe Ying Zhang, Guo Yang He, Zhi Qing Yuan, Na Li

**Affiliations:** 1 Department of Pathology, Xinxiang Medical University, Xinxiang, Henan 453003, P.R. China.; 2 Department of Physiology and Neurobiology, Xinxiang Medical University, Xinxiang, Henan 453003, P.R. China.; 3 Department of Radiology, The First Affiliated Hospital of Zhengzhou University, Zhengzhou, Henan 450000, P.R. China.; 4 School of Laboratory Medicine, Xinxiang Medical University, Xinxiang, Henan 453003, P.R. China.; 5 Xinxiang Key Laboratory of Immunoregulation and Molecular Diagnostics, Xinxiang, Henan 453003, P.R. China.

**Keywords:** Breast cancer, drug resistance, *EPAS1*, miR-152-3p, Paclitaxel

## Abstract

**Background:** Paclitaxel plays a pivotal role in the chemotherapy of breast cancer, but resistance to this drug is an important obstacle in the treatment. It is reported that microRNA-152-3p (miR-152-3p) is involved in tamoxifen resistance in breast cancer, but whether it is involved in paclitaxel resistance in breast cancer remains unknown.

**Materials and methods:** We examined the expression of miR-152-3p in breast cancer tissues and cells by qRT-PCR. After transfecting paclitaxel-resistant MCF-7/TAX cells with miR-152-3p mimics, we analyzed the function of miR-152-3p in these cells by MTT assay and flow cytometry. We screened the target gene, endothelial PAS domain-containing protein 1 (*EPAS1*), using bioinformatics analysis and verified it with the dual luciferase reporter gene experiment. The relationship between *EPAS1* and miR-152-3p and their roles in paclitaxel resistance of breast cancer were further investigated using RNA interference and transfection techniques.

**Results:** The expression of miR-152-3p in normal breast tissues and cells was markedly higher than that in breast cancer. Overexpression of miR-152-3p decreased the survival rate and increased the apoptosis rate and sensitivity of MCF-7/TAX cells to paclitaxel. We confirmed that *EPAS1* is the target of miR-152-3p and is negatively regulated by this miRNA. Moreover, transfection with EPAS1 siRNA enhanced the susceptibility and apoptosis rate of MCF-7/TAX cells to paclitaxel. Co-transfection of miR-152-3p mimics and *EPAS1* increased paclitaxel sensitivity and apoptosis induced by the drug.

**Conclusion:** miR-152-3p inhibits the survival of MCF-7/TAX cells and promotes their apoptosis by targeting the expression of *EPAS1*, thereby, enhancing the sensitivity of these breast cancer cells to paclitaxel.

## Introduction

Breast cancer is a heterogeneous neoplasm that originates in the glandular epithelium of the breast. This common malignant tumor threatens the physical and mental health of women worldwide 1-3. By 2050, the global incidence of breast cancer in women is predicted to increase by 3.2 million per year 4. The current strategy for treating breast cancer typically combines surgery with various complementary treatments, such as hormone therapy, chemotherapy, and targeted therapy. Among these methods, chemotherapy is a very important component of systematic treatment. However, resistance to drugs is the leading cause of failure of chemotherapy against breast cancer. Resistance of breast cancer to paclitaxel and other chemotherapeutic drugs has been shown to be related to epigenetic changes, imbalance of multiple signaling pathways, and gene mutations 5,6. However, the exact mechanism underlying chemoresistance remains unclear. Paclitaxel is a chemotherapeutic drug that is extensively used to treat various cancers. It prevents the segregation of chromosomes during mitosis by inhibiting the disassembly of microtubule polymers and stabilizing them, thereby, resulting in the induction of apoptosis. Paclitaxel has shown significant efficacy in the treatment of breast cancer, particularly triple-negative breast cancer. However, resistance to this drug is frequently observed. Because strategies for overcoming the resistance to paclitaxel can further potentiate its effect against breast cancer, it is important to investigate the mechanisms underlying this resistance.

MicroRNAs (miRNAs) are a group of endogenous small (20-25-nucleotides long), non-coding single stranded RNAs that play crucial roles in cell differentiation and biological development, as well as in the development of several diseases. The modulation of miRNA expression has been implicated in the regulation of tumor cell proliferation, apoptosis, differentiation, and metastasis 7-9. Moreover, the role of miRNAs in the development of drug resistance in breast cancer is crucial 10-12. In recent studies, miR-152 was found to be downregulated in numerous tumors, including gastric cancer, prostate cancer, and ovarian cancer, and was shown to function as a tumor suppressor 13-15. More importantly, miR-152 is involved in tamoxifen resistance in estrogen receptor-positive breast cancer 16. These studies suggest that miR-152 has a pivotal role in tumor formation and chemotherapy resistance. However, the involvement of this miRNA in the development of paclitaxel resistance in breast cancer remains unclear.

It has been reported that the abnormal expression of EPAS1 is closely related to the resistance of non-small cell lung cancer to tyrosine kinase inhibitors (TKIs) as well as to multidrug resistance in gastric cancer 17-18. In previous studies, our research group confirmed that the abnormal expression of endothelial PAS domain-containing protein 1 (*EPAS1*) is related to the proliferation, invasion, and angiogenesis of breast cancer, and participates in the chemoresistance of this cancer. Based on bioinformatics analysis performed using the TargtScan, Pictar, Pita, and Miranda software, we predicted that *EPAS1* is the putative target of miR-152-3p. In the present study, we examined the role of miR-152-3p in the development of paclitaxel resistance in breast cancer, and further explored whether targeting of *EPAS1* by this miRNA is involved in such resistance.

## Materials and Methods

### Human tissue collection

We collected 30 pairs of clinical samples (cancerous and normal) from breast cancer patients (nonspecific invasive breast carcinoma, NSIBC) undergoing surgical treatment in the Oncology Department of the First Affiliated Hospital of Zhengzhou University (Zhengzhou, China). The breast cancer and normal tissues (>5 cm from the edges of cancer tissues) were frozen in liquid nitrogen immediately after sampling. All the patients signed informed consent forms. No patient had undergone treatment, including radiotherapy, chemotherapy, or hormone therapy.

### Cell culture

MCF-7 cells were purchased from the Shanghai Cell Bank of Chinese Academy of Sciences; MCF-10A and MCF-7/TAX cells were purchased from the American Type Culture Collection (Manassas, VA, USA). MCF-7 and MCF-10A cells were cultured in RPMI 1640 medium containing 10% fetal bovine serum. MCF-7/TAX cells were cultured in RPMI 1640 medium containing 1.0 µM paclitaxel to maintain their resistant phenotype. The cells were cultured in a humidified incubator at 37 °C in an atmosphere of 5% CO_2_.

### Cell treatment

MiR-152-3p mimics, control mimics, EPAS1 siRNA, and siRNA control were purchased from Gene Pharma (Shanghai, China). EPAS1 overexpression and EPAS1 overexpression control vectors were purchased from Ribobio (Guangzhou, China). The miR-152-3p mimics (80 nM), miR-152-3p mimic control, EPAS1 siRNA, and EPAS1 siRNA control were transfected into MCF-7/TAX cells, generating the miR-152-3p, NC, si-EPAS1, and si-NC cell groups, respectively. MCF-7/TAX cells were co-transfected with miR-152-3p mimics and EPAS1 overexpression vector and were named as the miR-152-3p+EPAS1 and miR-152-3p+Vector groups, respectively. After 6 h of transfection, RPMI-1640 medium containing 10% fetal bovine serum (penicillin and streptomycin free) was added, and the cells were further cultured for 24 h.

### Real-time quantitative PCR

Total RNA from tissues and cells was extracted with TRIzol (Invitrogen, Carlsbad, CA, USA) and reverse transcribed using a reverse transcription kit (TaKaRa, Shiga, Japan), according to the manufacturer's instructions. The relative expression of target RNA in tissues and cells was determined by quantitative real-time polymerase chain reaction (qRT-PCR) using a SYBR Green test kit (TaKaRa). The optimized reaction conditions were as follows: denaturation at 95 °C for 5 min, followed by 40 cycles of denaturation at 95 °C for 30 s, annealing at 60 °C for 30 s, and extension at 72 °C for 15 s. The U6 gene was used as an internal control. The relative mRNA expression was calculated by the 2^-∆∆CT^ method 19.

### MTT assay

After transfection or co-culture with different concentrations of paclitaxel, the cell suspension (100 μL) was seeded into 96-well plates at a density of 6.5 × 10^3^ cells/well and incubated overnight in a 37 °C incubator. Subsequently, 10 μL of 10 mg/mL MTT was added to each well, and the plate was incubated at 37 °C for 4 h. Thereafter, the culture medium was aspirated and 150 μL of dimethyl sulfoxide was added to each well. After complete dissolution of the purple crystals that had formed, the absorbance was measured at 570 nm using a microplate reader. The average value of readings from three replicate experiments was used to calculate the half maximal inhibitory concentration (IC_50_).

### Detection of apoptosis by flow cytometry

Apoptosis was determined using an apoptosis detection kit (Beyotime, Shanghai, China). Briefly, the MCF-7/TAX cells in each group were trypsinized, and 400 µL of 1× binding buffer was added to prepare a cell suspension containing 1 × 10^6^ cells/mL. Annexin V-FITC (5 µL) and propidium iodide (PI; 5 µL) were added to 100 µL cell suspension, and allowed to react in the dark for 15 min at 20 °C. The apoptotic ratio of cells was determined by flow cytometery (BD Biosciences, Franklin Lakes, NJ, USA).

### Dual luciferase reporter gene assay

Based on a search of related literature and different databases (TargtScan, Pictar, Pita, Miranda), we predicted that *EPAS1* is the target gene of miR-152-3p. The sequence of the 3′-untranslated region (UTR) of *EPAS1* was amplified and inserted into the reporter gene vector, *pGL3*, which was designated as the *EPAS1* wt plasmid. A mutated fragment of 3′-UTR of *EPAS1* was also amplified and inserted into the *pGL3* vector, which was designated as the *EPAS1* mut plasmid. miR-152-3p mimics and miR-152-3p NC were co-transfected into MCF-7/TAX cells with the EPAS1 wt and EPAS1 mut plasmids, respectively. After 36 h, the cells were lysed with Passive Lysis Buffer (Promega, Madison, WI, USA) and the supernatant was collected. Luciferase activity was detected using a dual luciferase reporter gene detection kit (Promega) following the manufacturer's instructions. Relative luciferase activity was calculated using the formula: relative luciferase activity = fluorescence value using Renilla luciferase/fluorescence value using firefly luciferase.

### Western blot analysis

Protein was extracted from the cells by lysing them in lysis buffer (Abcam, Cambridge, UK). The concentration of proteins in the lysate was determined using a BCA protein assay kit (Keygen, Nanjing, China). Proteins were resolved by SDS-PAGE and subsequently transferred onto a polyvinylidene fluoride membrane. The membrane was blocked in blocking buffer with shaking at 20 °C for 1 h. It was then washed twice with phosphate-buffered saline containing Tween 20 and incubated with primary antibodies against EPAS1 and GAPDH (Abcam) for 15 h at 4 °C. Thereafter, the membrane was washed twice with phosphate-buffered saline containing Tween 20 and incubated with goat anti-mouse IgG for 2 h at 20 °C. Protein expression was detected using ImageJ software (NIH, Bethesda, MD, USA).

### Statistical analysis

SPSS 2.1 software (SPSS, Inc., Chicago, IL, USA) was used for statistical analysis of experimental data, and 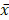
±s was used for the measurements. One-way analysis of variance was used to compare the differences among groups. The difference between two groups was analyzed by the SNK-q test. *P <* 0.05 indicates that the difference was statistically significant.

## Results

### Overexpression of miR-152-3p sensitizes MCF-7/TAX cells to paclitaxel and increases the apoptotic rate

#### Expression of miR-152-3p in human breast cancer tissues and cells

**Figure 1:**
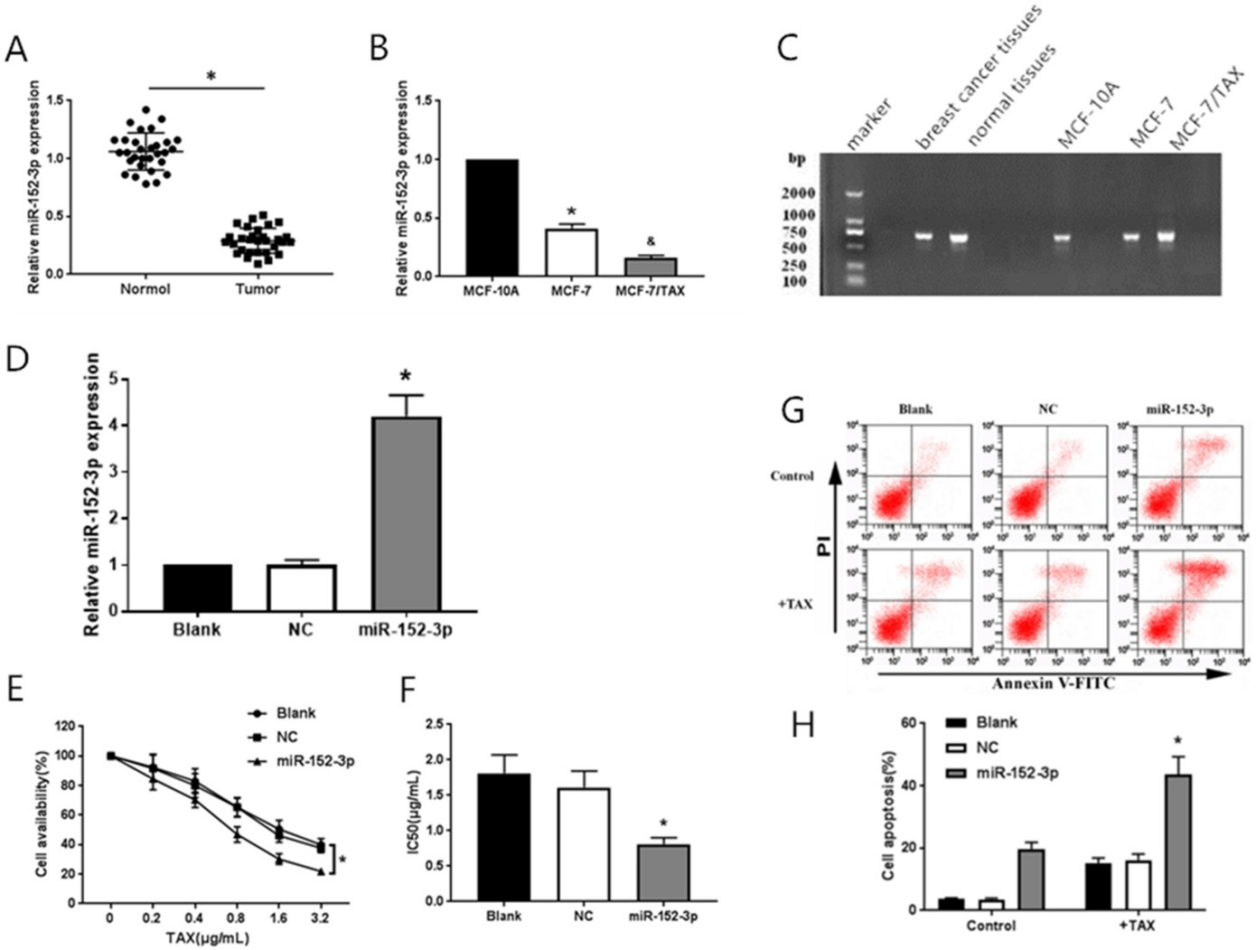
Effect of miR-152-3p over expression on paclitaxel induced MCF-7/TAX cells. **A:** Expression of miR-152-3p in human breast cancer tissues was lower than in normal tissues. **B:** Expression of miR-152-3p in MCF-7 cells and paclitaxel resistant MCF-7/TAX cells was significantly lower than in normal MCF-10A cells. *P < 0.05, vs. normal tissues and MCF-10A cells; ^&^*P <* 0.05, vs. MCF-7 cells. **C:** RNA electrophoresis of miR-152-3p isolated from breast cancer tissues and cells. **D:** Expression of miR-152-3p in MCF-7/TAX cells was significantly increased by transfection of miR-152-3p mimics. **E-F:** Paclitaxel, at concentrations of 0, 0.2, 0.4, 0.8, 1.6, and 3.2 µg/mL, was used to treat MCF-7/TAX cells in each group; the survival rate and IC_50_ of MCF-7/TAX cells in the miR-152-3p group was significantly lower than of the NC group. **G-H:** Treatment of MCF-7/TAX cells with paclitaxel at a concentration of 0.4 µg/mL; overexpression of miR-152-3p enhanced paclitaxel-induced apoptosis of MCF-7/TAX cells. *P < 0.05.

To study the function of miR-152-3p in human breast cancer, qRT-PCR was used to detect 30 pairs of breast cancer tissues and paracancerous tissues from 30 patients with nonspecific invasive breast carcinoma. The results showed that the expression of miR-152-3p was low in human breast cancer relative to that in normal tissues. The expression of miR-152-3p in breast cancer cells was further detected by qRT-PCR. The results showed that the expression of miR-152-3p in MCF-7 cells and paclitaxel-resistant MCF-7/TAX cells was significantly lower than that in normal MCF-10A cells, and the expression of miR-152-3p in MCF-7/TAX cells was lower than that in MCF-7 cells (Fig. 1A-C). This indicates that the expression of miR-152-3p is low in breast cancer tissues and cells.

#### Expression of miR-152-3p in breast cancer cells after transfection with miR-152-3p mimics

MiR-152-3p mimics and negative control mimics were transfected into MCF-7/TAX cells. The transfection efficiency was detected by qRT-PCR, 48 h after transfection. The expression of miR-152-3p in MCF-7/TAX cells in the miR-152-3p group was significantly higher than in the NC group (Fig. 1D). This indicated that MCF-7/TAX cells overexpressing miR-152-3p were successfully constructed.

#### Effect of overexpression of miR-152-3p on paclitaxel resistance in MCF-7/TAX cells

To further explore the role of miR-152-3p in paclitaxel resistance of breast cancer, paclitaxel at concentrations of 0, 0.2, 0.4, 0.8, 1.6, and 3.2 µg/mL were used to interfere with MCF-7/TAX cells in each group, and MTT assay was used to detect cell viability. The survival rate of MCF-7/TAX cells in miR-152-3p group was significantly lower than that in the NC group (Fig. 1E). Based on the cell survival rate, the IC_50_ of each group was calculated. The results showed that the IC_50_ values of MCF-7/TAX cells in the blank, NC, and miR-152-3p groups were 1.81 ± 0.26, 1.61 ± 0.23, and 0.81 ± 0.09 µg/mL, respectively. The IC_50_ in the miR-152-3p group was lower than in the NC group, but the differences between the NC and blank groups were not significant (Fig. 1F). The results showed that overexpression of miR-152-3p can make MCF-7/TAX cells more sensitive to paclitaxel.

#### Effect of miR-152-3p overexpression on paclitaxel-induced apoptosis in MCF-7/TAX cells

MCF-7/TAX cells were induced with paclitaxel at a concentration of 0.4 µg/mL for 48 h. Flow cytometry was used to detect apoptosis in each group. The results showed that the apoptosis rates for MCF-7/TAX cells not treated with paclitaxel in the blank, NC, and miR-152-3p groups were 3.64% ± 0.40%, 3.55% ± 0.39%, and 19.74% ± 2.12%, respectively; the apoptotic rates for MCF-7/TAX cells treated by paclitaxel in the blank, NC, and miR-152-3p groups were 15.22% ± 1.62%, 16.18% ± 1.87%, and 43.69% ± 5.61%, respectively (Fig. 1G, H). These results indicate that overexpression of miR-152-3p can enhance paclitaxel-induced apoptosis of MCF-7/TAX cells.

### miR-152-3p regulates the expression of *EPAS1*

#### Expression levels of miR-152-3p and EPAS1 are negatively correlated

**Figure 2:**
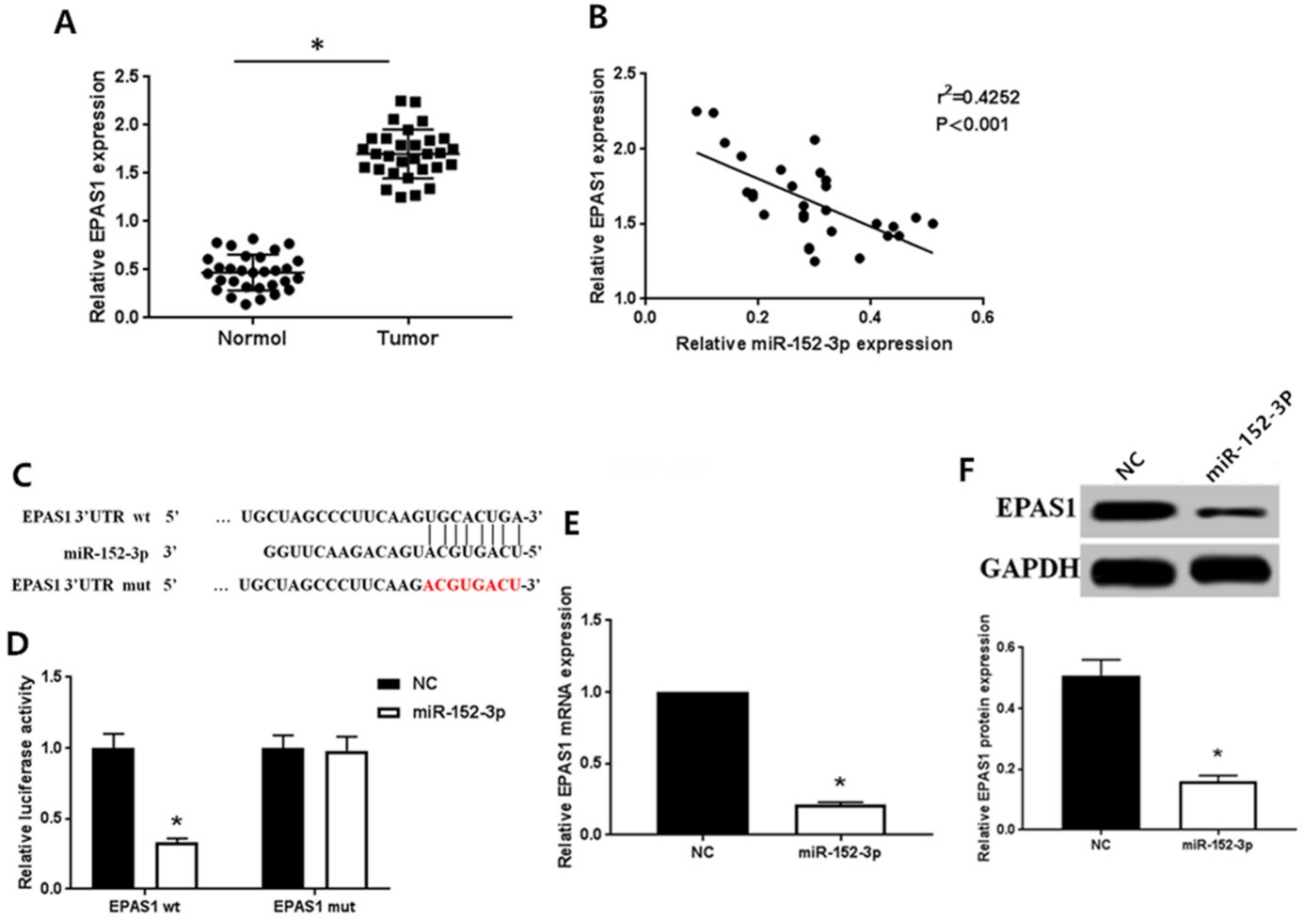
MiR-152-3p targeted regulation of *EPAS1* expression. **A:** Expression of EPAS1 in breast cancer is higher than in normal tissues, as detected by qRT-PCR. **B:** Spearman correlation analysis showed that the expression of miR-152-3p was negatively correlated with the expression of *EPAS1*. **C:** Schematic diagram of miR-152-3p and *EPAS1* target binding sites predicted by TargetScan. **D:** MiR-152-3p regulates the expression of *EPAS1* by binding to the 3′-UTR site of *EPAS1*. **E-F:** Compared to the NC group, the expression level of *EPAS1* mRNA and protein in MCF-7/TAX cells in the miR-152-3p group was significantly reduced. *P < 0.05.

As detected by qRT-PCR, the expression of *EPAS1* in normal tissues was remarkably lower than in breast cancer tissues (Fig. 2A), which contrasted the expression pattern of miR-152-3p. Spearman correlation analysis showed that the expression of miR-152-3p was inversely correlated with that of *EPAS1* (Fig. 2B).

#### EPAS1 is the direct target of miR-152-3p

Bioinformatics analysis (TargtScan, Pictar, Pita, Miranda) to identify the gene targeted by miR-152-3p revealed a specific binding site for miR-152-3p in *EPAS1* (Fig. 2C). We used dual luciferase reporter assay to further verify that *EPAS1* is a potential target of miR-152-3p. The results showed that the relative luciferase activity of the vector cells co-transfected with miR-152-3p mimics and EPAS1 wild type 3′-UTR was significantly inhibited compared with that of cells co-transfected with miR-152-3p NC and EPAS1 wild type 3′-UTR in MCF-7/TAX cells. However, when miR-152-3p mimics or miR-152-3p NC and *EPAS1* mutant 3′-UTR were co-transfected, the relative luciferase activity of the cells did not change significantly (Fig. 2D). The results showed that miR-152-3p could regulate the expression of EPAS1 by binding to the 3′-UTR site of *EPAS1*.

#### miR-152-3p targeting regulates the expression of EPAS1

To further explore the regulation of *EPAS1* expression by miR-152-3p, we detected the levels of *EPAS1* mRNA and protein in paclitaxel-resistant cells overexpressing miR-152-3p by qRT-PCR and western blot analysis. The expression of *EPAS1* in MCF-7/TAX cells in the NC group was markedly higher than in the miR-152-3p group both at the mRNA and protein levels (Fig. 2E, F). This demonstrates that miR-152-3p negatively regulates the expression of *EPAS1*.

### miR-152-3p targeting of *EPAS1* regulates paclitaxel resistance in breast cancer cells

To detect whether the mechanism underlying the miR-152-3p-mediated increase in chemosensitivity of MCF-7/TAX cells to paclitaxel involves the regulation of *EPAS1*, we performed RNA interference and functional complementation experiments.

#### Transfection with EPAS1 siRNA increases the sensitivity of MCF-7/TAX cells to paclitaxel

**Figure 3:**
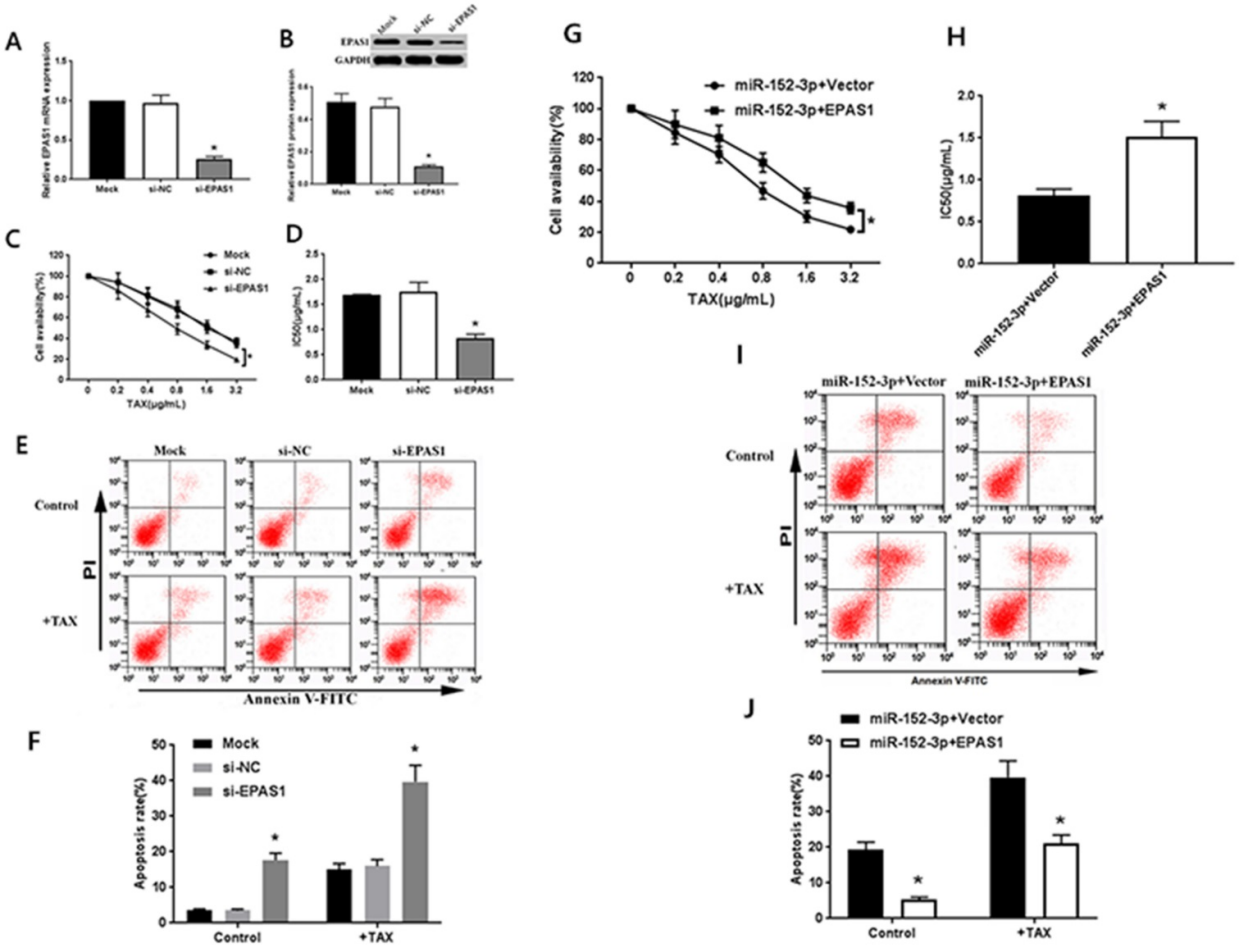
Targeting of *EPAS1* by miR-152-3p regulates paclitaxel resistance in breast cancer cells. **A-B:** Expression of *EPAS1* mRNA and protein in the si-EPAS1 group was significantly lower than in the si-NC group after transfection of EPAS1 siRNA. **C-D:** Paclitaxel, at concentrations of 0, 0.2, 0.4, 0.8, 1.6, and 3.2 µg/mL, was used to treat MCF-7/TAX cells in each group; the survival rate and IC_50_ of MCF-7/TAX cells in the si-EPAS1 group was significantly lower than in the si-NC group. **E-F:** Treatment of MCF-7/TAX cells with paclitaxel at a concentration of 0.4 µg/mL; knockdown of *EPAS1* increased paclitaxel-induced apoptosis of MCF-7/TAX cells. **G-H:** Co-transfection of miR-152-3p mimics and EPAS1 overexpression or control into MCF-7/TAX cells, compared to in the miR-152-3p+Vector group; the cell survival rate and IC_50_ of the miR-152-3p+EPAS1 group were increased. **I-J:** Overexpression of *EPAS1* can reverse the apoptosis of MCF-7/TAX cells induced by the overexpression of miR-152-3p and inhibit the apoptosis of MCF-7/TAX cells. *P < 0.05.

The specific EPAS1 siRNA was transferred to MCF-7/TAX cells. After 48 h of transfection, the results of qRT-PCR and western blot analysis showed that EPAS1 siRNA could significantly inhibit the expression of EPAS1 mRNA and protein in MCF-7/TAX cells (Fig. 3A, B). After the treatment of MCF-7/TAX cells with different concentrations of paclitaxel, the results of MTT assay showed that the cell survival rate in the si-EPAS1 group was significantly lower than that in the si-NC group, and there was no significant difference between the si-NC and mock groups (Fig. 3C). The IC_50_ values of MCF-7/TAX cells in the mock, si-NC, and si-EPAS1 groups were 1.69 ± 0.17, 1.75 ± 0.19, and 0.83 ± 0.08 µg/mL, respectively (Fig. 3D). The IC_50_ of MCF-7/TAX cells in the si-NC group was apparently higher than that in the si-EPAS1 group. This suggests that inhibition of *EPAS1* can increase the chemosensitivity of MCF-7/TAX cells to paclitaxel.

#### Knockdown of EPAS1 promotes paclitaxel-induced apoptosis of MCF-7/TAX cells

We knocked down *EPAS1* and detected the apoptosis rate for each group. The results of flow cytometry analysis showed that the apoptosis rates for MCF-7/TAX cells not treated with paclitaxel in the mock, si- NC, and si-EPAS1 groups were 3.55% ± 0.39%, 3.54% ± 0.36%, and 17.64% ± 1.96%, respectively; the apoptotic rates for MCF-7/TAX cells treated with paclitaxel in the mock, si-NC, and si-EPAS1 groups were 15.08% ± 1.59%, 16.01% ± 1.77%, and 39.72 ± 4.59%, respectively (Fig. 3E, F). These results suggest that knockdown of *EPAS1* can increase the apoptosis of MCF-7/TAX cells induced by paclitaxel.

#### Overexpression of EPAS1 reverses the sensitization of miR-152-3p mediated MCF-7/TAX cells to paclitaxel

To confirm that the regulation of *EPAS1* by miR-152-3p is involved in the resistance of MCF-7/TAX cells to paclitaxel, miR-152-3p mimics and EPAS1 overexpression or control vectors were co-transfected into MCF-7/TAX cells for functional complementation experiments. Experimental data showed that compared with miR-152-3p+vector group, the cell viability and IC_50_ of miR-152-3p + EPAS1 group were increased (Fig. 3G, H). It indicated that the overexpression of *EPAS1* reversed the increased sensitivity of MCF-7/TAX cells to paclitaxel induced by overexpression of miR-152-3p and promoted paclitaxel resistance of MCF-7/TAX cells.

#### Overexpression of EPAS1 reverses the apoptosis of MCF-7/TAX cells induced by miR-152-3p

The results of flow cytometry showed that the apoptosis rates of MCF-7/TAX cells in the miR-152-3p + vector and miR-152-3p + EPAS1 groups without paclitaxel intervention were 19.26% ± 2.21% and 5.39% ± 0.64% respectively; the apoptosis rates of MCF-7/TAX cells in the miR-152-3p + vector and miR-152-3p + EPAS1 groups with paclitaxel intervention were 39.72% ± 4.59% and 21.13% ± 2.32%, respectively (Fig. 3I, J). It is suggested that overexpression of *EPAS1* can reverse the apoptosis of MCF-7/TAX cells induced by overexpression of miR-152-3p and inhibit the apoptosis of MCF-7/TAX cells. It is suggested that miR-152-3p can promote apoptosis of MCF-7/TAX cells by inhibiting *EPAS1*.

## Discussion

Paclitaxel is the first-line chemotherapeutic drug for treating malignant tumors. However, in breast cancer, the emergence of drug resistance is an important cause of chemotherapy failure. Many studies have revealed several types of miRNA dysregulation in breast cancer, which are involved in resistance to chemotherapy 20-22. In oral squamous cell carcinoma, researchers have shown that miR-152 can suppress the proliferation, invasion, and metastasis of cancer cells by targeting c-MET 23. It was previously reported that miR-152 represses the transformation and angiogenesis of breast cancer by targeting DNMT1 24. It is reported that human leukocyte antigen G (HLA-G) can make tumor escape immune surveillance and is highly expressed in a variety of tumors, such as gastric cancer, renal cell carcinoma, and breast cancer 25-27. MiR-152 has a strong regulatory function on HLA-G and downregulates the expression of HLA-G by targeting its 3′-UTR 26,28. Therefore, miR-152 is thought to be a tumor suppressor in many cancers. Furthermore, Chen et al. showed that miR-152 reduces tamoxifen resistance by downregulating the expression of ALCAM in breast cancer cells 16. Moreover, there have been reports that miR-152 enhances adriamycin resistance by targeting SPIN1 29. Accumulating evidence shows that miR-152 greatly affects drug resistance in breast cancer. However, little is known about its role in paclitaxel resistance of breast cancer and about the mechanisms underlying any such role.

In this study, we detected the expression of miR-152-3p in breast cancer cells and tissues. In addition, we found that miR-152-3p showed the lowest expression in MCF-7/TAX cells. This indicates that miR-152-3p is involved in the occurrence and development of breast cancer and paclitaxel resistance. To further confirm the effect of miR-152-3p on the resistance of breast cancer cells to paclitaxel, we performed MTT and flow cytometry analyses. We observed that the overexpression of miR-152-3p effectively reversed paclitaxel resistance in breast cancer cells.

According to previous reports, miR-152-3p targets ADAM17 and TMEM97 in non-small cell lung cancer and prostate cancer, inhibiting the activity of tumor cells 30,31. This suggests that miR-152-3p exerts multiple biological functions by targeting different target genes. In a previous study, we used bioinformatics software to predict the potential target binding sites for miR-152-3p in *EPAS1*.

EPAS1, also known as hypoxia inducible factor 2α, is absent in normal tissues or is expressed at low levels; however, it is widely expressed under hypoxic conditions 32,33. EPAS1 can participate in various physiological and pathological processes, such as bone marrow hematopoiesis and vascular growth. It is also involved in the occurrence, progression, and angiogenesis of tumors, as well as in their antiapoptotic behavior. In the tumor microenvironment, because of the formation of irregular capillaries, blood flow is slow; however, because of the increase in oxygen demand of tumor cells and for other reasons, there is a lack of oxygen 34. By activating the gene expression program, EPAS1 starts the adaptive response of cells to the hypoxic environment 35. It has been reported that EPAS1 is expressed abnormally in several tumors, including breast cancer, and has a large effect on the regulation of metastasis and progression of breast cancers 36,37. Particularly, high expression of EPAS1 is closely related to cisplatin resistance in lung adenocarcinoma and multidrug resistance in stomach cancer 38,18. These results suggest that *EPAS1* is related to the resistance of tumors to chemotherapy. In a previous study, we confirmed that abnormal expression of EPAS1 is involved in tumor proliferation, invasion, and angiogenesis, and is related to the resistance of breast cancer to chemotherapy; however, whether EPAS1 has other functions in breast cancer and its upstream regulatory molecules are unclear.

Taibi et al. showed that mir-148a attenuates the overexpression of *EPAS1* in intestinal inflammation 39. There may be some relationship between *EPAS1* and miR-152-3p. To further decipher the relationship between miR-152-3p and EPAS1 and their effects on the progression of breast cancer, we used qRT-PCR, Spearman's correlation, and western blot analyses to show that miR-152-3p may play a negative regulatory role in the expression of *EPAS1*.

To further explore whether the increased sensitivity of MCF-7/TAX to paclitaxel induced by miR-152-3p is related to the regulation of *EPAS1* expression, we performed interference and complementation experiments. The results showed that transfection with EPAS1 siRNA inhibited the expression of *EPAS1* in MCF-7/TAX cells and increased the sensitivity of these cells to paclitaxel. This suggests that EPAS1 is related to paclitaxel resistance in breast cancer cells. Moreover, overexpression of *EPAS1* counteracted the increased sensitivity of MCF-7/TAX cells to paclitaxel caused by overexpression of miR-152-3p.

In conclusion, our study advances the understanding of paclitaxel resistance of breast cancer cells, which seriously influences its therapeutic efficacy. We confirmed that miR-152-3p inhibits the survival of MCF-7/TAX cells and promotes their apoptosis by targeting *EPAS1* expression, thereby, enhancing the sensitivity of these cells to paclitaxel. We also provide a new target for paclitaxel resistance in breast cancer.
